# Impact on the scape of *Farfugium japonicum* var. *japonicum* (Asteraceae) under strong wind conditions based on morphological and mechanical analyses

**DOI:** 10.3389/fpls.2024.1407127

**Published:** 2024-08-06

**Authors:** Masayuki Shiba, Shuma Arihara, Shiori Harada, Tatsuya Fukuda

**Affiliations:** ^1^ Graduate School of Integrative Science and Engineering, Tokyo City University, Setagata, Tokyo, Japan; ^2^ Department of Science and Engineering, Tokyo City University, Setagata, Tokyo, Japan

**Keywords:** *Farfugium japonicum*, mechanical properties, petiole, scape, wind conditions, thigmomorphogenesis

## Abstract

Adaptation of *Farfugium japonicum* (L.) Kitam. var. *japonicum* (Asteraceae) to the strong wind environment of coastal areas has been shown to reduce lamina size and shorten petioles; however, their effects on other traits of this species remain unknown. Our morphological analyses showed that shortening of the scape of this species is correlated with shortening of the petiole in coastal areas. The results suggested that when the height of the scapes became higher than that of the petioles, the wind stress on the scapes became stronger and their growth was suppressed. Therefore, the populations in coastal areas with strong winds had significantly shorter scapes than inland populations, and the height of petioles and scapes in the coastal populations were correlated. Further mechanical analysis by three-point bending tests revealed that the scapes had higher strength than the petioles. This species is evergreen and can produce new leaves regardless of the season, even if it loses its leaves by strong winds; however, because scapes only develop above ground for a limited period of the year, the loss of the scapes by strong winds has a significant impact on reproduction in that year. Therefore, even though the scapes were stronger than the petiole, shortening the scapes plays an important role in reducing strong wind stress in coastal areas.

## 1 Introduction

The adaptations of each plant organ to the environment have been central to the study of plant diversification. Fitness is an important means of evaluating plant adaptation to various environmental factors (Buonaiuto and Wolkovich, 2021; Creux et al., 2021), and serves as an index for evaluating the adaptability of plant morphological and anatomical characteristics in specific environments and the contribution of individual plants to the production of the next generation (Strauss, 1997). For example, plant groups that inhabit riverside areas have narrower leaves to resist and/or avoid flashing floods after heavy rains (Imaichi and Kato, 1992; Yamada et al., 2011; Ueda et al., 2012; Yokoyama et al., 2012; Ohga et al., 2012a; Matsui et al., 2013; Shiba et al., 2021; Shiba and Fukuda, 2024), and plants in serpentine areas exhibit severe morphological and anatomical limitations related to plant growth (Brady et al., 2005; Hayakawa et al., 2012; Ohga et al., 2012b; Kumekawa et al., 2013; Shiba et al., 2022a; 2022b). Wind is one of the most ubiquitous environmental stresses on many levels in natural ecosystems (Mylo and Speck, 2023), and the avoidance of wind stress using flexible and easily reconfigurable structures can be an alternative strategy that contributes to maintaining fitness (Biddington, 1986; Anten et al., 2009). How plants adapt or acclimate to such variable external forces depends on the intensity and frequency of stress as well as on plant structures. Some previous studies have indicated that plant responses to wind typically lowered photosynthesis (Ennos, 1997; Burgess et al., 2016), decreased leaf area (Anten et al., 2010; Shiba et al., 2023) and entailed inhibition of stem elongation and increased in stem diameter to stabilize the plant mechanically (Jaffe and Forbes, 1993; Telewski and Pruyn, 1998; Coutand and Moulia, 2000; Anten et al., 2010; Bang et al., 2010; Niez et al., 2019), and wind stress affects various aspects of plant life history such as development, growth, pollination, seed dispersal, insect herbivory and reproductive yield (Ennos, 1997; Wilcock and Neiland, 2002; Hodges et al., 2004; Fernandez and Hilker, 2007; Turbill, 2008; Takizawa et al., 2022; Shiba et al., 2022c, 2023).

**Figure 1:**
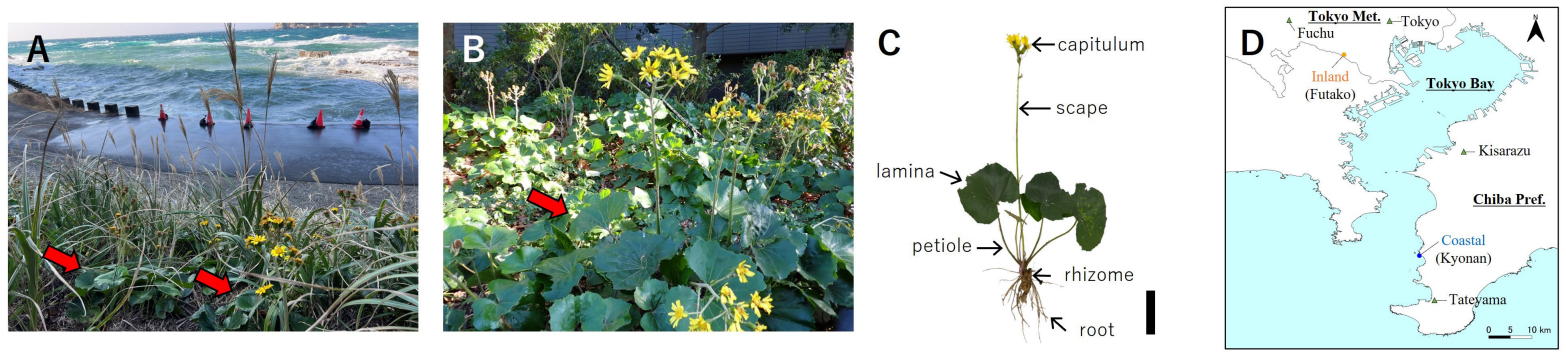
*Farfugium japonicum* var. *japonicum* (Asteraceae) at the Kyonan coast **(A)**, and Futako inland **(B)**. Arrows indicate *F japonicum*. Silhouette of *F japonicum* **(C)**, scale bar = 10 cm. Sampling sites of *F japonicum* **(D)**. Regarding sampling sites, orange squares indicate Futako inland and blue circles indicate Kyonan coast. The location of the AMeDAS is indicated by a green triangle.

Coastal areas are characterized by open, sandy, or rocky habitats with weakly developed soils. These characteristics can lead to the modification of the morphologies and plant species growing in the coastal regions of the world as they need to be adapted to an environment in which drought and wind strongly affect plant growth (Greenway and Munns, 1980). Some previous studies have been conducted on adaptation to drought (Tunala et al., 2012; Kumekawa et al., 2013; Ohga et al., 2013; Sunami et al., 2013; Takizawa et al., 2022; Shiba et al., 2022c, 2023) and wind stress (Meguro and Miyawaki, 1994; Shiba et al., 2023) in the coastal areas. Shiba et al. (2023) indicated that the leopard plant *Farfugium japonicum* (L.) Kitam. var. *japonicum* (Asteraceae) (**Figure 1**), an evergreen perennial herb that occurs from the margin of inland forests to near the coast in Japan (Koyama, 1995), has avoided an increase in wind speed outdoors by reducing the lamina area and petiole length per petiole cross-sectional area without mechanical changes of their petioles to the wind by using populations near meteorological recording stations of the Automated Meteorological Data Acquisition System (AMeDAS) of the Japan Meteorological Agency. However, this study was only part of the strategy against wind stress by linking the leaf morphology of *F. japonicum* and meteorological information in the field (Shiba et al., 2023), and it is questionable whether the reductions in these traits alone are sufficient to adapt *F. japonicum* var. *japonicum* to wind stress in coastal areas. The evaluation of other morphological traits except for these traits is also important for understanding the environmental adaptations of this plant. In this study, the traits of *F. japonicum* var. *japonicum* should be the focus of future studies. For example, Khan et al. (2016) reported that root architecture plays an important role in the anchorage capability to withstand toppling and overturning caused by wind loading. Wind not only induces thigmomorphogenesis in plants by altering root architecture (Chehab et al., 2009), but also improves the anchorage of plants by strengthening the development of roots (Stokes et al., 1995; Danjon et al., 2005; Tamasi et al., 2005; Štofko and Kodrík, 2008). In addition, Onoda and Anten (2011) claimed that strong winds could increase root biomass allocation, and other previous studies have indicated that wind affects root growth and biomass allocation to roots (Nicoll and Ray, 1996; Cleugh et al., 1998; Poorter et al., 2012; Gardiner et al., 2016; Feng et al., 2019). By considering these studies, does strong winds also affect the root architecture as in lamina and petiole of *F. japonicum* var. *japonicum*? However, it remains unclear whether *F. japonicum* var. *japonicum* in coastal areas increases root biomass allocation.

Floral display is fundamental to plant fitness, which can affect the pollinator visitation rate and also total seed production (Bell, 1985), and is, therefore, under strong stabilizing selection, ensuring phenotypic homeostasis even under conditions of environmental and genetic perturbation (Givnish, 2002). *F. japonicum* var. *japonicum* have long scapes from 30 to 75 cm that support the capitulum, blooming yellow flowers from October to December, and an approximately 30 cm long petiole with a lamina approximately 10 cm long and 20 cm wide at the tip of the petiole all year round (Koyama, 1995). Although their radical leaves remain exposed to wind stress for a long period after appearing on the ground, the scape appears on the ground for a shorter period than the petioles. Does the difference in the emergence period reflect the difference in the adaptive traits between radical leaves and scapes? The scape extending from the rhizomes grows independently of the petioles, so even if the petioles become shorter with the same stiffness owing to the influence of strong winds over a long period, does this have a little effect on the morphological and mechanical traits of the scape? To answer this question, an investigation into the morphological and mechanical comparisons of the scapes and petioles, as well as scapes of coastal and inland populations of *F. japonicum* var. *japonicum* is needed in order to clarify the differences in petioles and scapes in response to strong winds in coastal areas. Several studies have been conducted on the strength of inflorescences using the model plant *Arabidopsis thaliana* (L.) Heynh (Yoshida et al., 2019; Nakata et al., 2020), the effect of strong winds on inflorescences or scapes remains unclear.

Many traits should be analyzed related to the adaption to the strong wind environment of coastal areas, in addition to lamina size and petiole length of *F. japonicum* var. *japonicum*, which have already been reported by Shiba et al. (2023). However, it is difficult to comprehensively analyze the various traits of *F. japonicum* var. *japonicum*. In general, plants are limited by the resources available in their natural environment; it is impossible for plants to achieve the best in all aspects, and they often have to adapt to their environment by making compromises (Funk and Vitousek, 2007). Therefore, as the resource allocation of one organ increases, that of another decreases, meaning that the cost of one organ can be measured by the resource consumption of the other (Reznick, 1985; Jösson and Tuomi, 1994; Wise and Abrahamson, 2017). Only by effectively adjusting resource allocation can plants achieve their optimal performance in complex environments and provide competitive advantages for survival and reproduction (Funk and Vitousek, 2007). Elucidating whether resource allocation is involved in shape differences would provide the basis for avoidance or tolerance to strong wind stress. Therefore, the evaluation of the above-mentioned traits of *F. japonicum* var. *japonicum* is one way to demonstrate the adaptation process in the strong wind environments of coastal areas by comparing their resource allocation patterns. The aim of this study is to clarify the effects of strong winds on the above-ground morphology of *F. japonicum* var. *japonicum* and its avoidance strategies from both morphological and mechanical aspects.

## 2 Materials and methods

The collection sites and nearby weather stations and their annual average wind speed are presented in in **Figure 1D**, **Table 1**. In the present study, *Farfugium japonicum* was collected from field populations in coastal and inland areas. The wind speed values are higher on the oceanside (Tateyama 3.40 m s-1, Kisarazu 2.88 m s-1, Tokyo 2.75 m s-1, Fuchu 1.71 m s-1), and the coastal sampling site Kyonan, located between Tateyama and Kisarazu, is assumed to have higher wind speed values than the inland sampling site Futako, which is located between Tokyo and Fuchu. Nomura et al. (2010) showed that there had no genetic differentiation among populations of *F. japonicum* based on some sequences of Internal Transcribed Spacer (ITS) of nuclear DNA and intergenic regions between *trnL* and *trnF* and between *trnH* and *psbA* of chloroplast DNA. The study was conducted from November to December during the flowering period of *F. japonicum*. Almost all roots were dug during collection. Another condition for collection was that the leaf blade and petiole were largely intact, and individuals of various sizes were collected. The field survey and sampling were conducted in areas where there were no collection restrictions under Japanese law.

**Table 1:** Sampling sites in this study.

Name	Locality	Longitude Latitude	Habitat	Soil condition
Sampling				
Kyonan	Kyonan, Awa District, Chiba Pref.	35.06.08.927 139.49.35.760	coastal	humus
Futako	Kaminoge, Setagaya-ku, Tokyo Met.	35.36.35.819 139.38.08.915	inland	black soil
AMeDAS		Annual average wind speed (2023-Jan.-Dec.)		
Tateyama	Nagasuka, Tateyama City, Chiba Pref.	3.40 m s-1		
Kisarazu	Zyouzaiminami, Kisarazu City, Chiba Pref.	2.88 m s-1		
Tokyo	Kitanomaru Park, Chiyoda-ku, Tokyo Met.	2.75 m s-1		
Fuchu	Shinmachi, Fuchu City, Tokyo Met.	1.71 m s-1		

**Figure 2:**
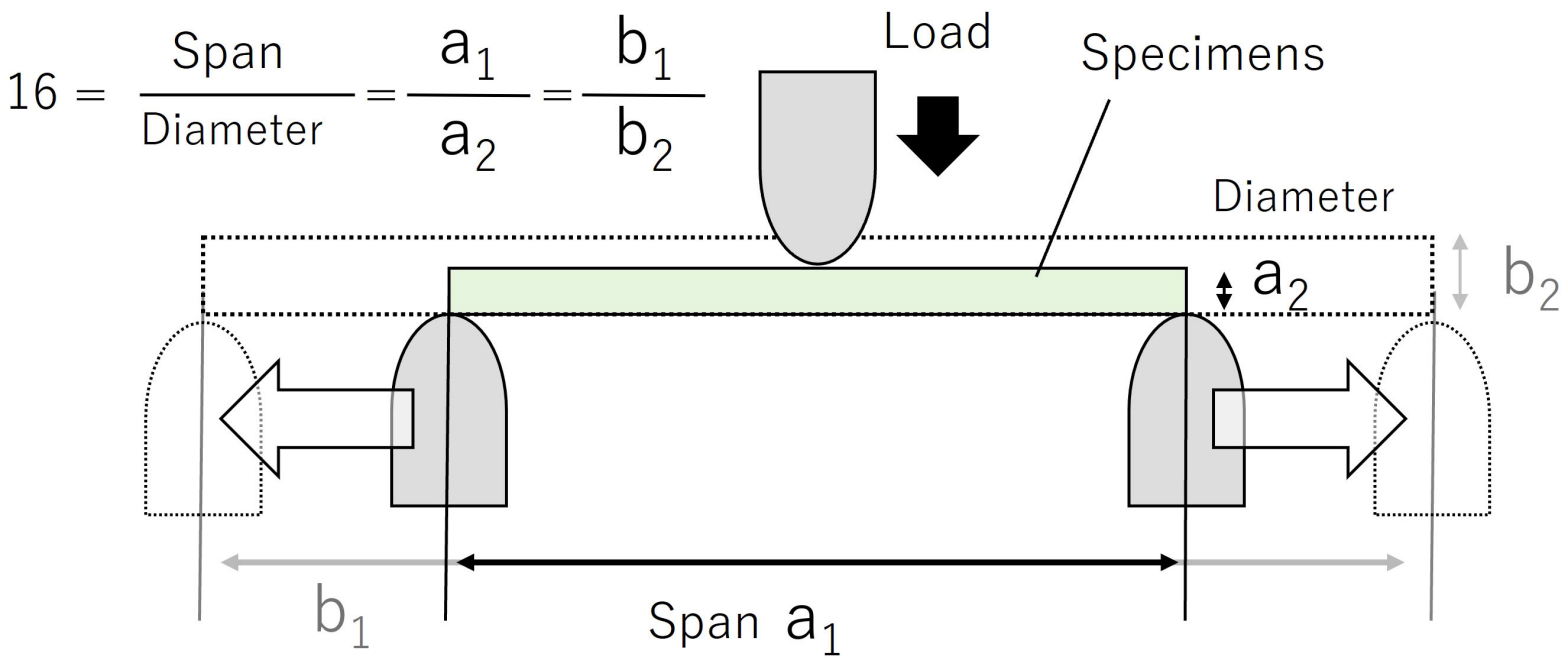
Set-up diagram for a three-point bending test. Move the support pin so that the span/diameter ratio is 16. The load is applied to the center of the specimen at constant velocity.

Name corresponds to that given in **Figure 2**.

### 2.1 Morphological analyses

For morphological analyses, the lamina area was analyzed using the graphic software Fiji (ImageJ Version 1.53e) for radical leaves. Petiole length *l*_petiole_ and scape length *l*_scape_ were measured using digital calipers CD-15APX (Mitutoyo Corp., Kawasaki-shi, Kanagawa, Japan). Cross-sectional area A was calculated using the following formula to approximate it as an ellipse:


$$\begin{array}{c} A=\frac{\pi ab}{4} \end{array}$$

where a and b are the long and short diameters of the petiole and scape cross sections, respectively, measured using the digital calipers. The basal cross-sectional area *A*_base_ was calculated in the same manner as described in the aforementioned equation. In addition, the cross-sections of petioles and scapes were visually confirmed to be elliptical in shape and were compared for similar elliptical shapes based on the ratio of long/short diameter.

The number of radical leaves per plant was determined. The number of capitulae per scape were counted.

### 2.2 Dry mass analysis of organs

In general, the net increase in dry matter is the sum of the products of photosynthesis and mineral uptake minus the amount lost in respiration, leaching, guttation, leaf and twig abscission, and death of roots, and much of the discussion on the fitness costs associated with increased competitive ability has concentrated on the effects of dry matter partitioning within the plant (e.g., Poorter et al., 2012). Therefore, many studies have compared the dry weight of each organ to compare the allocation patterns, and we also used this method.

After morphological analyses, the cells were dried in an incubator FS-405 (ADVANTEC Co., Ltd., Tokyo, Japan) set at 75°C for at least 72 hours. The dried flowers (scapes and capitula), radical leaves (lamina and petiole), rhizomes, and roots were immediately weighed [g] using an analytical balance AG204 (Mettler-Toledo, Tokyo, Japan).

Dry mass per unit volume of petioles and scapes was calculated using the following equation:


$$\begin{array}{c} Dry\;mass\;per\;unit\;volume=\;\frac{Dry\;mass}{V_{fresh}} \end{array}$$

where *V_fresh_* is the volume of the petioles or scapes measured by the dry mass.

*V_fresh_* was calculated using the following equation:


$$V_{fresh}=(\pi ab/4)\times h$$

where *h* is the height of petioles or scapes. The dry mass per unit volume is an indicator of the cell-wall content of the analyzed organ. The relationship between the dry mass per unit volume and cross-sectional area was analyzed.

In addition, the flowers and leaves were divided into the aboveground part organs and rhizomes and roots were divided into below ground part organs, and the above/below ground part dry mass ratio was analyzed.

### 2.3 Mechanical analyses of petioles and scapes

A three-point bending test was used for the mechanical testing (**Figure 2**). Samples used for mechanical testing were processed fresh within 12 hours of collection. The test setup consisted of a tabletop tensile and compression testing machine MCT-1150 (A&D Company, Ltd., Tokyo, Japan) and either an R5 bending test fixture JM-B1-500N(A&D Company, Ltd., Tokyo, Japan) or an R2 bending test fixture JM-B-500N (A&D Company, Ltd., Tokyo, Japan) if the average sample diameter was less than 3 mm. The test speed was set to 10 mm min-1. The termination criterion for the test was set when the displacement of the upper fixture reached 3 mm. The specimens had to be cut for mechanical testing, and the average diameter was measured before the test on the non-tapered portion of the petiole or scape, and the length of the specimen and the distance between spans were set so that the ratio of span/average diameter was “16”. This span-to-diameter ratio condition was expected to yield mechanical results when the general bending theory was applied (Shah et al., 2017).

The mechanical tests in this study were conducted on simply supported beams, so the values of bending stress *σ* and strain *ϵ* were calculated using the following equations:


$$\begin{array}{c} \sigma =\frac{M}{Z}=\frac{PL}{4Z} \end{array}$$

and


$$\begin{array}{c} \epsilon =\frac{48\delta \sigma I}{PL^{3}}\times 100 \end{array}$$

where *P* is the load measured in the three-point bending test, *M* is bending moment at concentrated load at mid-span, *L* is the span length, *Z* is the section modules, *δ* is the deflection measured in the three-point bending test, and *I* is the second moment of area. In this study, the dimensionless strain ϵ is converted to %.

The following equations were used to calculate the elliptical section coefficient *Z* and the axial second moment of area *I*:


$$\begin{array}{c} Z=\frac{\pi ab^{2}}{32} \end{array}$$

and


$$\begin{array}{c} I=\frac{\pi ab^{3}}{64}. \end{array}$$

The bending modulus of elasticity *E* was calculated by multiplying the slope of the approximately straight linear part of the stress–strain curve by 100. In addition, the flexural rigidity *EI* was calculated using the following equation:


$$\begin{array}{c} EI=E\times I. \end{array}$$

Data on specimen sizes are presented in **Supplementary Table 1**.

### 2.4 Anatomical analysis

Cross-sectional tissues of petioles and scapes were observed. A manual microtome CMK (Kenis, Ltd., Osaka city, Osaka, Japan) was used to prepare cross-sectional sections of approximately 20 µm thickness. One section of the petiole and scape bases was stained with a 0.05% toluidine blue O solution (pH 7.0) for 1 min and then washed thoroughly with distilled water. Toluidine blue O stains lignified tissue, corky tissue, tannins blue-green, and non-lignified cell walls red-purple (Sakai, 1973). Stained sections were photographed using an optical microscope CX43 (Olympus Corp., Tokyo, Japan) equipped with a digital microscope camera Moticam X3-12V (Shimadzu Rika Corp., Tokyo, Japan).

### 2.5 Statistical analysis

Statistical analyses were performed using the GNU R 4.3.3 software (R Core Team, 2024). These analyses involved various statistical tests to compare the different groups and examine the relationships between variables. Specifically, we utilized the Shapiro-Wilk test (p<0.05) to assess the normality of the data distribution. When the assumption of normality was violated, we employed the Mann-Whitney *U* test (p<0.05) to compare two independent groups or the Kruskal-Wallis test (p<0.05) to compare four independent groups. Welch’s *t*-test (p<0.05) was used when data followed a normal distribution. Additionally, we conducted an analysis of covariance to examine the relationship between the variables while controlling for covariates, we conducted an analysis of covariance (ANCOVA). The correlation coefficient and the coefficient of determination when fitting a linear regression were examined. These statistical methods were selected based on the characteristics of the data and the specific hypotheses tested.

## 3 Results

### 3.1 Morphological analysis

**Figure 3:**
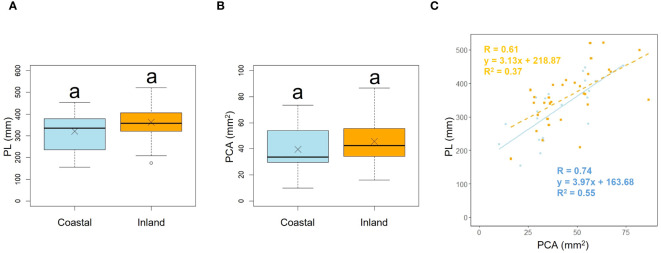
Comparison of the petiole length (PL) **(A)** and petiole cross-sectional area (PCA) **(B)** between coastal and inland populations. Following the results of the Shapiro-Wilk test (*p*< 0.05), the petiole length was compared using the Mann-Whitney *U* test (*p* = 0.22), while the petiole cross-sectional area was compared using Welch’s *t*-test (*p* = 0.19). Sample numbers of coastal and inland, n_coastal_ = 21, n_inland_ =31. Box-whisker plots, median, quartiles, outliers; crosses indicate the position of the mean. Same letters indicate no statistically significant difference. PCA vs PL for coastal (blue circle and solid line) and inland (orange square and dashed line) plant samples (PCA: Habitat*p* = 0.46) **(C)**.

Comparison of the longest petiole length (PL) and basal petiole cross-sectional areas (PCA) of individuals indicated no significant difference between the coastal and inland areas (PL; *p* = 0.22, mean; coastal = 320.85, inland = 362.28: PCA; *p* = 0.19, mean; coastal = 39.60, inland = 45.76), indicating that petiole length and cross-sectional area were comparable between the two populations (**Figures 3A, B**, **Table 2**). We investigated whether the influence of petiole cross-sectional area on petiole length varied depending on the habitat. Analysis of covariance indicated no correlation between petiole cross-sectional area and habitat (*p* = 0.46). However, significant differences were observed in the intercepts (*p*< 0.001: **Figure 3C**).

**Table 2:** Morphological measurements and above/below ground plant part mass ratios (mean ± standard error) of petioles and scapes in *Farfugium japonicum*.

	Coastal				Inland				
petioles									
length (mm)	332.67 ± 20.43 (n = 22)		a		358.89 ± 15.47 (n = 30)		a	
cross-sectional area (mm^2^)	39.60 ± 3.60 (n = 21)		a		45.76 ± 2.96 (n = 31)		a	
scapes									
length (mm)	419.12 ± 19.53 (n = 30)		b		571.06 ± 17.71 (n = 32)		a	
cross-sectional area (mm^2^)	52.70 ± 6.87 (n = 21)		a		46.53 ± 2.90 (n = 30)		a	
above/below ground plant part mass ratios (-)	0.58 ± 0.08 (n = 21)		a		0.63 ± 0.07 (n = 26)		a	
	Petiole coastal		Petiole inland		Scape coastal		Scape inland	
Long/short diameter ratios (-)	1.06 + 0.01	a	1.06 ± 0.009	a	1.07 ± 0.02	a	1.04 ± 0.007	a

Different small letters indicate significant difference according to *t*-test (p<0.05).

**Figure 4:**
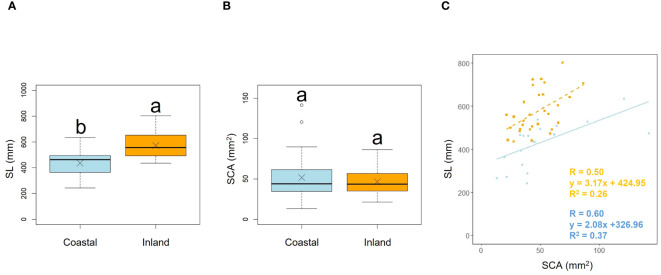
Comparison of scape length (SL) **(A)** and scape cross-sectional area (SCA) **(B)** between coastal and inland populations. Following the results of the Shapiro-Wilk test (*p*< 0.05), the scape length was compared using Welch’s *t*-test (*p*< 0.001), while the scape cross-sectional area was compared using the Mann-Whitney *U* test (*p* = 0.90). Sample numbers of coastal and inland, n_coastal_ = 21, n_inland_ =30. Box-whisker plots, median, quartiles, outliers; crosses indicate the position of the mean. Same letters indicate no statistically significant difference. SCA vs. SL for coastal (blue circle and solid line) and inland (orange square and dashed line) plant samples (SCA: Habitat, *p* = 0.36) **(C)**.

**Figure 5:**
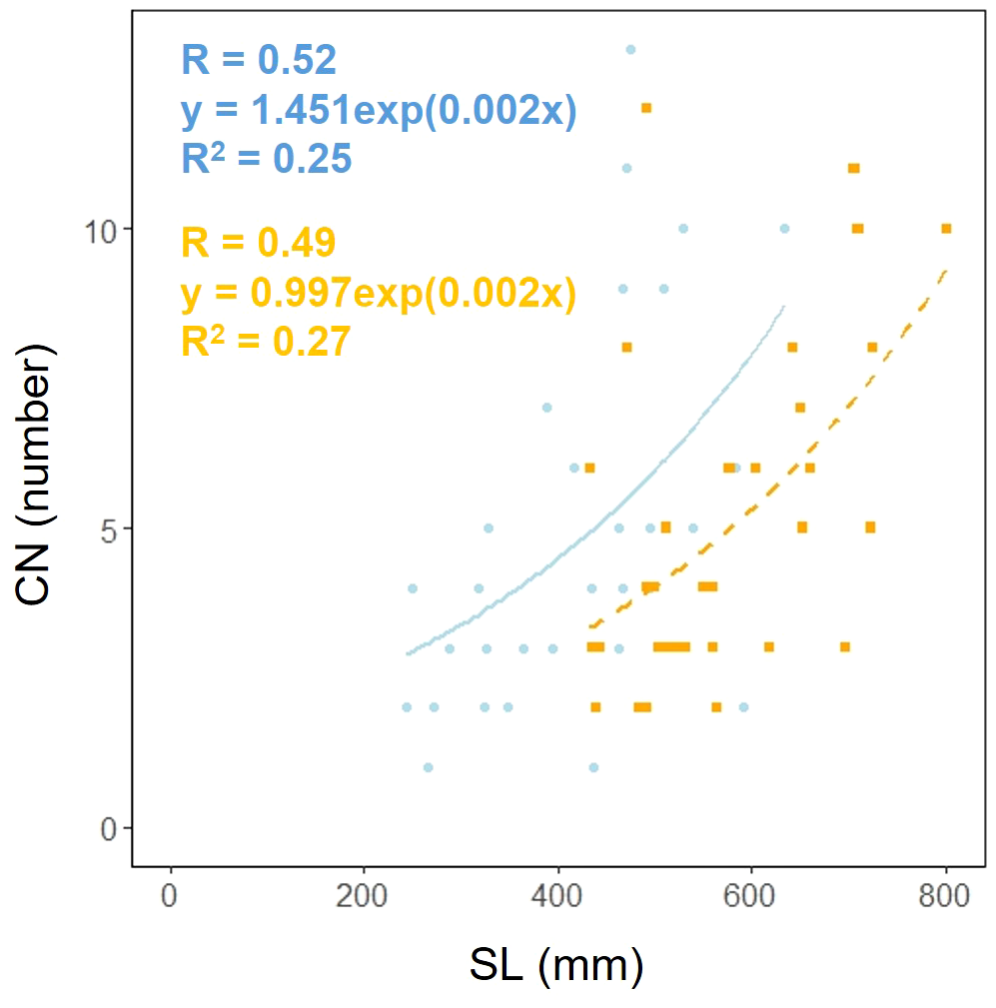
Capitulum number (CN) vs. scape length (SL) for coastal (blue circle and solid line) and inland (orange square and dashed line) plant samples (SL: Habitat, *p* = 0.80). Sample numbers of coastal and inland, n_coastal_ = 30, n_inland_ =32.

Scape length (SL) and basal cape cross-sectional area (SCA) were compared. The results indicated that scape length was significantly smaller in the coastal region (SL; *p*< 0.001, mean; coastal = 434.54, inland = 572.64: **Figure 4A**, **Table 2**). However, there was no significant difference in the cross-sectional area of the scape, indicating a similar thickness (SCA; *p* = 0.90, mean; coastal = 51.69, inland = 46.52: **Figure 4B**, **Table 2**). We investigated whether the influence of scape cross-sectional area on scape length varied depending on the habitat. Analysis of covariance indicated no correlation between cross-sectional area and habitat (*p* = 0.36). However, significant differences were observed in the intercept (*p*< 0.001: **Figure 4C**). We examined whether the effect of scape length on capitulum number (CN) varied depending on habitat. The results of comparing the fitting of a linear regression model with a non-linear regression model indicated that the non-linear regression model was a better fit. Analysis of covariance results indicated no correlation between scape length and habitat (*p* = 0.80: **Figure 5**).

**Figure 6:**
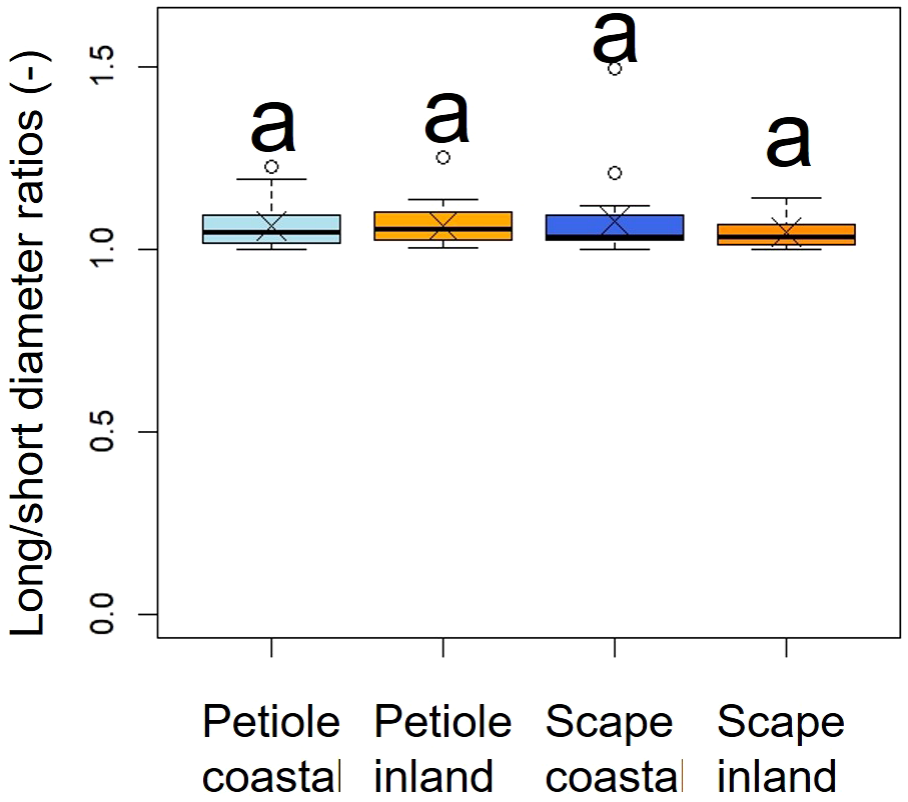
Comparison of long/short diameter ratios in the cross sections of petioles and scapes. The Kruskal-Wallis test indicated no significant difference (*p* = 0.49); indicated by same letters. Crosses indicate the position of the mean. Petiole; n_coastal_ = 21, n_inland_ = 31, Scape; n_coastal_ = 21, n_inland_ = 30. Box-whisker plots, mean, quartiles, outliers; crosses indicate the position of the mean. Same letters indicate no statistically significant difference.

A comparison of long/short diameter ratios in the cross-sections of petioles and scapes indicated no significant differences between the two populations (*p* = 0.49, mean; petiole coastal = 1.06, inland = 1.06, scape coastal = 1.07, inland = 1.04), indicating a similar elliptical shape (**Figure 6**, **Table 2**).

**Figure 7:**
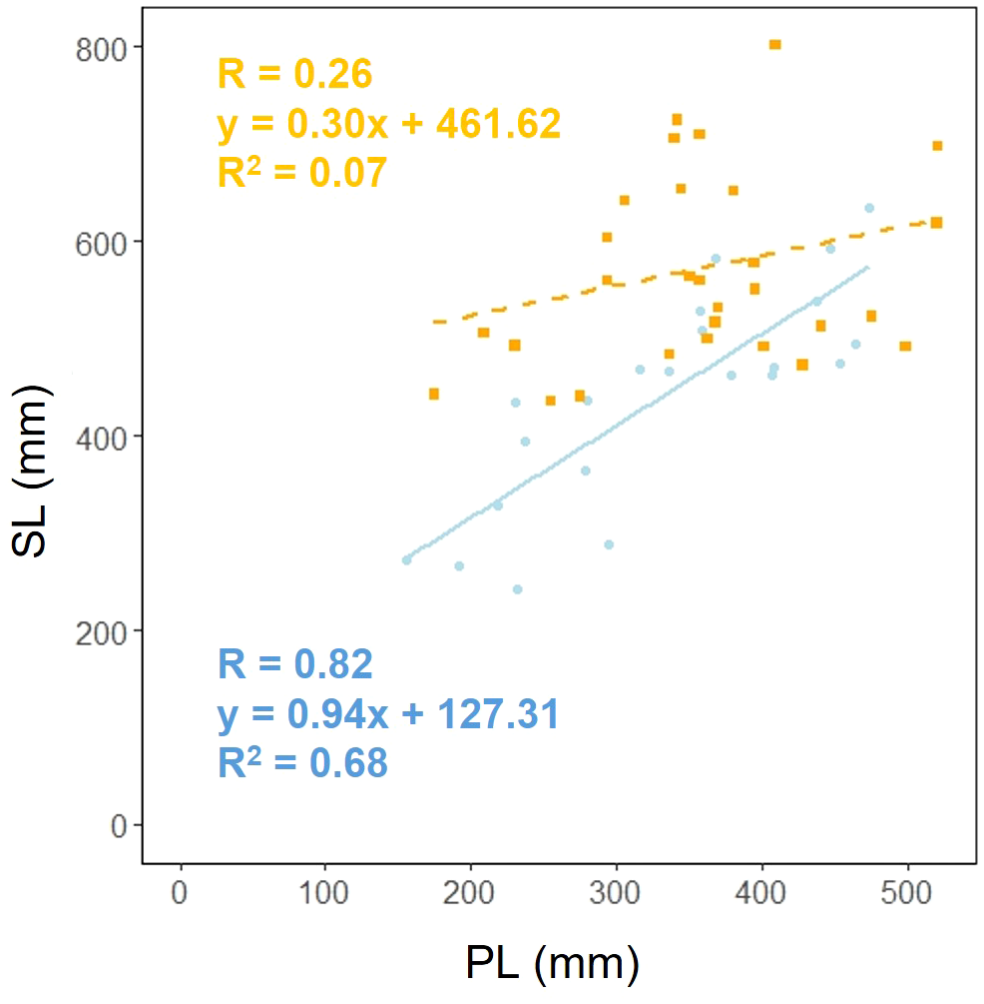
Petiole length (PL) vs. scape length (SL) for coastal (blue circle and solid line) and inland (orange square and dashed line) plant samples (PL: Habitat, *p<* 0.05). Sample numbers of coastal and inland, n_coastal_ = 22, n_inland_ = 30.

We investigated whether the effect of scape length (SL) on petiole length (PL) varied depending on the habitat. Analysis of covariance showed that there was a correlation between petiole length and habitat (*p*< 0.05), indicating that different habitats influence the relationship between petiole length and petiole length (**Figure 7**).

### 3.2 Relationship between total dry mass and each morphological trait

**Figure 8:**
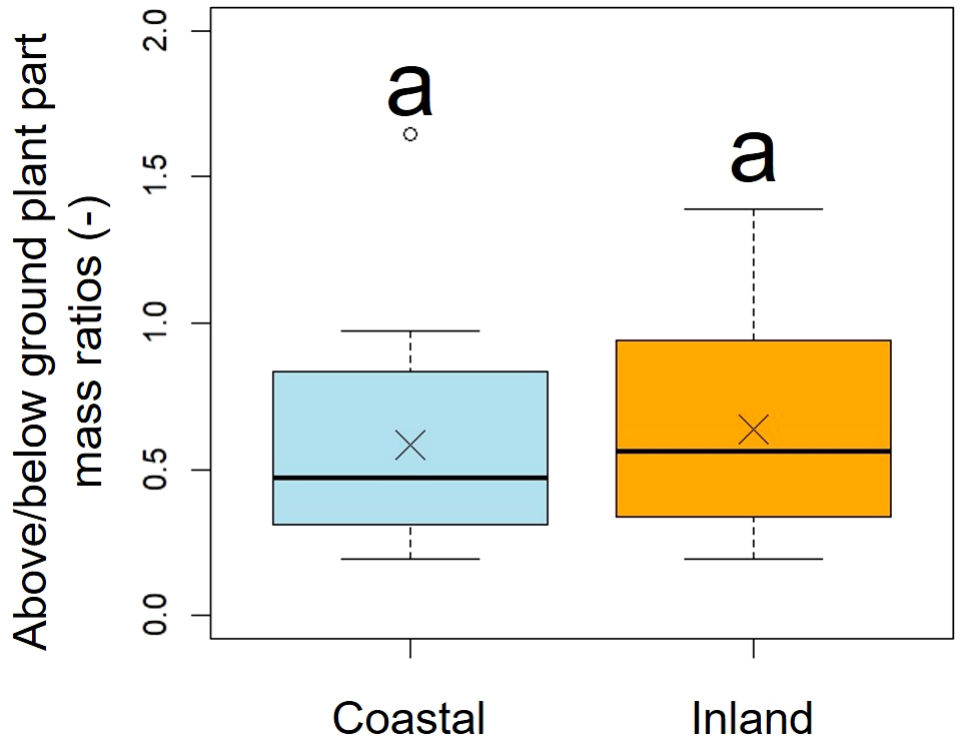
Comparison of the above/below ground plant part mass ratios. Followed the results of the Shapiro-Wilk test (*p*< 0.05), the above/below ground plant part mass ratios were compared using the Mann-Whitney *U* test (*p* = 0.51). Sample numbers of coastal and inland, n_coastal_ = 21, n_inland_ =26. Box-whisker plots, median, quartiles, outliers; crosses indicate the position of the mean. Same letters indicate no statistically significant difference.

A comparison of above/below ground part mass ratios indicated no significant difference between the two populations (*p* = 0.51, mean; coastal = 0.58, inland = 0.63), indicating similar dry mass ratios (**Figure 8**, **Table 2**).

**Figure 9:**
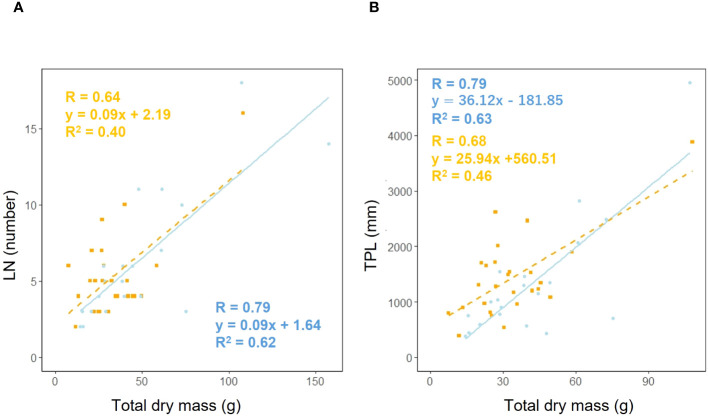
Leaf number (LN) vs. total dry mass **(A)** for coastal (blue circle and solid line) and inland (orange square) plant samples (total dry mass: Habitat, *p* = 0.90). Sample numbers of coastal and inland, n_coastal_ = 21, n_inland_ =26. Total petiole length (TPL) vs. total dry mass **(B)** for coastal (blue circle and solid line) and inland (orange square and dashed line) plant samples (total dry mass: Habitat, *p* = 0.24). Sample numbers of coastal and inland, n_coastal_ = 20, n_inland_ =26.

We examined whether the effect of leaf number (LN) on total dry mass varied depending on habitat. Analysis of covariance showed no correlation between total dry mass and habitat (*p* = 0.90), suggesting that the effect of habitat on the relationship between total dry mass and leaf number is additive (**Figure 9A**). We investigated whether the effect of total petiole length (TPL) on total dry mass varied depending on habitat. Analysis of covariance showed no correlation between total dry mass and habitat (*p* = 0.24), indicating that the effect of habitat on the relationship between total dry mass and petiole length is additive (**Figure 9B**).

**Figure 10:**
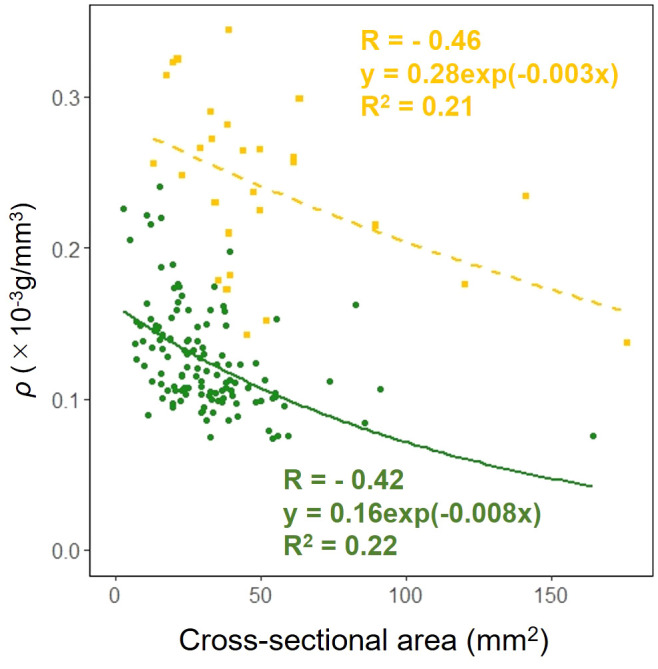
Cell wall mass per unit petiole and scape volume *ρ* vs base cross-sectional area of petiole (green circle and solid line) and scape (yellow square and dashed line) (cross-sectional area: Habitat, *p* = 0.90). Sample numbers of petiole and scape, n_petiole_=123, n_scape_=28.

We examined whether the effect of the cross-sectional area on the dry mass per unit volume *ρ* in the petioles and scapes varied depending on the organ. The results of comparing the fitting of a linear regression model with a non-linear regression model indicated that the non-linear regression model was a better fit. The exponential approximation formula was also the best fit for the non-linear regression model. Analysis of covariance results indicated no correlation between cross-sectional area and organ (*p* = 0.90: **Figure 10**).

### 3.3 Mechanical properties of petioles and scapes

**Figure 11:**
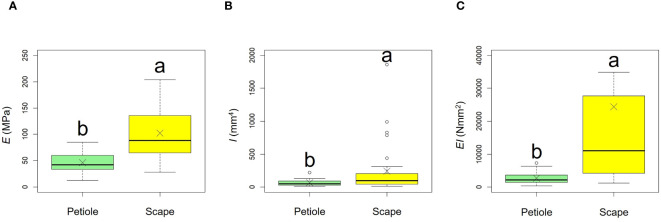
Comparison of the bending modulus of elasticity *E* **(A)**, axial second moment of area *I* **(B)**, and flexural rigidity *EI* **(C)** between petiole and scape. Following the results of the Shapiro-Wilk test (*p*< 0.05), the *E*, *I*, and *EI* were compared using the Mann-Whitney *U* test (*E*, *I*, *EI*: p<0.05). Sample numbers of petiole and scape, n_petiole_ = 35, n_scape_ = 33. Box-whisker plots, mean, quartiles, outliers; crosses indicate the position of the mean. Same letters indicate no statistically significant difference.

Comparative results of mechanical properties are shown in **Figure 11**, **Table 3**. A comparison of the *E* values of the scapes were significantly higher than those of the petioles (*p* < 0.001, mean; petiole = 45.90, scape = 102.00). A comparison of the *I* values of the scapes were significantly higher than those of the petioles (*p* < 0.01, mean; petiole = 62.26, scape = 238.59). A comparison of the *EI* values of the scapes were significantly higher than those of the petioles (*p* < 0.001, mean; petiole = 2678.84, scape = 24354.46).

**Table 3:** Mechanical properties (mean ± standard error) measured for the petioles and scapes in *Farfugium japonicum*.

	Petioles		Scapes	
Bending modulus of elasticity *E* (MPa)	45.91 ± 3.01 (n = 35)	b	103.11 ± 7.97 (n = 33)	a
Axial second moment of area *I* (mm^4^)	62.26 ± 7.36 (n = 35)	b	238.60 ± 65.57(n = 33)	a
Flexural rigidity *EI* (Nmm^2^)	2678.85 ± 301.85 (n = 35)	b	24354.46 ± 6345.63(n = 33)	a

Different small letters indicate significant difference according to *t*-test (p<0.05).

### 3.4 Histological structure on petiole and scape cross-section

**Figure 12:**
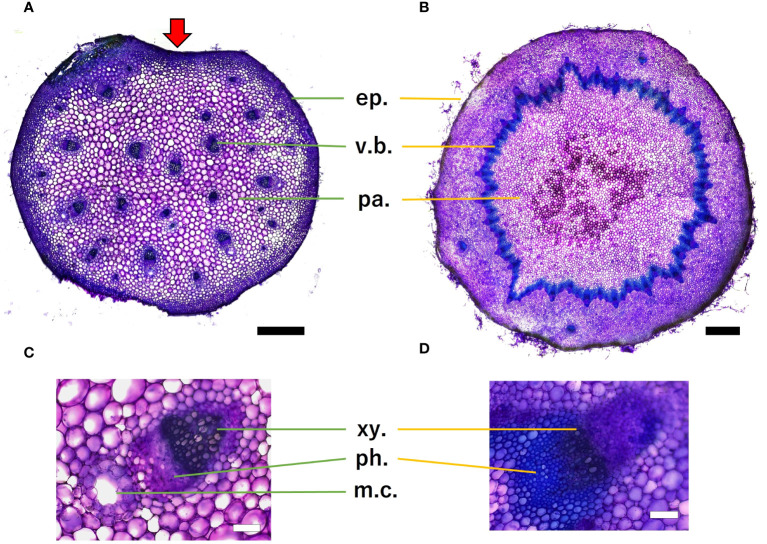
Microscopic images of petiole **(A)** and scape **(B)** cross-section, scale bar = 0.5 mm. Red arrows indicate micro canal-shaped grooves. Vascular bundles of petiole **(C)** and scape **(D)**, scale bar = 100 µm. The epidermis (ep.), the vascular bundle (v.b.), parenchyma (pa.), xylem (xy.), phloem (ph.), and mucilage channels (m.c.) are shown. Toluidine blue staining showed that blue-green stained lignified tissue and purple stained non-lignified cell walls.

Histological analysis revealed that the petiole stelar type was an atactostele (**Figure 12A**), and the scape stelar type was an eustele (**Figure 12B**). Mucilage channels were observed in petiole. Scape had several vascular arrangements outside of the annular vascular bundle. Toluidine blue staining revealed lignification near the epidermis and vascular bundles in both organs, while parenchyma was non-lignified. Within the vascular bundles, xylem stained blue more than phloem, indicating that it was more lignified.

In terms of cross-sectional shape, the present study assumed an elliptical shape for both organs in the analysis of mechanical properties, but cross-sectional observation of the petiole revealed a “micro canal-shaped grooves” that developed on the vertical side of the petiole relative to the adaxial side of the lamina.

## 4 Discussion

Plant growth responses to thigmomorphogenesis include changes in branch and foliar development, stem shape and mass, leaf size, branch size, and stem height, all of which are restricted by the mechanical action of wind (Telewski and Pruyn, 1998). In our study, similar to the findings of Shiba et al. (2023), there were no significant differences found in the morphological characteristics of the lamina and petiole between inland and coastal habitats (**Figures 3** **4**). However, significant differences were observed in scape length between habitats (**Figure 4A**), and differences in the distribution of data between habitats were evident in the relationship between scape cross-sectional area and scape length (**Figure 4C**). Perennial plants are comprised of individuals at various growth stages, and individual size and reproductive investment increase with growth (Samson and Werk, 1986). The number of flowers and inflorescences per individual can influence plant fitness by increasing pollinator attraction (Ohashi and Yahara, 1999; Makino et al., 2007). In addition, the taller the scape, the more capitula and flowers it can hold and the greater its influence on the frequency of flower visits by pollinators (Levin and Kerster, 1973; Pyke, 1981). In our study, there were no differences in the aboveground and below ground dry mass ratios of *F. japonicum* between the habitats (**Figure 8**). However, coastal *F. japonicum* exhibited shorter scape lengths, and also significant differences were observed in the distribution of data concerning the relationship between scape length and capitulum number, indicating that when attaching a similar number of capitulae, inland plants were able to position them higher than coastal plants (**Figure 5**). Furthermore, petiole length in the coastal populations of *F. japonicum* var. *japonicum* were correlated with those of the scape (**Figure 7**). The scapes of inland populations with low wind stress were significantly higher than those of coastal populations, regardless of petiole length. Why do the lengths of petioles and scapes correlate with strong wind stress in coastal areas? Nagashima and Hikosaka (2011) indicated that plants reduce their height growth to maintain similarity to neighboring plants, because artificially lifted individuals in the population have reduced plant height growth compared to others, contributing to the avoidance of strong wind stress, which has been reported in several plants since this study (Nagashima and Hikosaka, 2012; Masaki et al., 2013; Thomas et al., 2015; Gruntman et al., 2017). Based on these findings, if the escape lengths of *F. japonicum* var. *japonicum* far exceeded that of the petioles, this meant that the scape would be strongly affected by strong wind stress in coastal areas. This was alleviated by petioles and laminas, with the expectation that the risk of breakage of the scape would increase. Therefore, we considered that the length of the petioles and the scapes were correlated because the growth of the scapes repeatedly decreased owing to the influence of strong winds in coastal areas when the scapes exceeded the height of the petioles and laminas. Similar shortening of petioles and scapes under strong winds was expected to have similar mechanical strengths. Mechanical analysis of the scape and petioles is presented below.

*F. japonicum* var. *japonicum* increases the number of flowers and capitula to attract pollinators; however, this also increases the load on the scape as the tip of the scape becomes heavier by increasing number of them. In the present study, significant differences were observed in the distribution of data on the relationship between scape length and capitulum number. Specifically, when attaching a similar number of capitulae, inland plants were able to position themselves higher than coastal plants (**Figure 5**). However, our results do not provide insights into the specific arrangement of the capitula within the scape. Plant species have acquired unique strengths to support their aboveground organs (Shiba et al., 2024), and their resistance to breakage or overturning under strong wind conditions depends largely on structural modifications in terms of morphology, anatomy, and mechanical strength (Shiba et al., 2023). Mechanical studies have focused on agricultural crops and woody products (Gibson et al., 1988; Kokubo et al., 1989; Skubisz, 2001, 2002; Green et al., 2006; Lim et al., 2011; Christoforo et al., 2012; Slater and Ennos, 2013; Lemloh et al., 2014; Sekhon et al., 2020), and there are some studies on plant adaptation (Spatz et al., 1998; Anten et al., 2010; Hesse et al., 2020; Wolff-Vorbeck et al., 2021; Langer et al., 2022; Mylo et al., 2022; Shiba et al., 2023, 2024; Shiba and Fukuda, 2024). These previous studies have shown that analyses from a mechanical perspective are necessary not only for petioles, but also for scapes of *F. japonicum* var. *japonicum*. In particular, the maintenance of the scape during the flowering period of *F. japonicum* var. *japonicum* is important for the production of the next generation, and if scape is lost, the flowers and seeds of this species cannot be produced until the following year. Our results showed that the scapes of *F. japonicum* var. *japonicum* was stronger than in the petiole (**Figure 11**). The mechanism by which *F. japonicum* var. *japonicum* can increase the strength of scape through the petiole? The petioles and scapes of *F. japonicum* var. *japonicum* exhibited similar elliptical cross-sectional shapes (**Figure 6**). However, the axial second moment of the area, which depends on the diameter, was significantly higher in the scapes, indicating greater resistance to bending. Moreover, our results showed that the dry mass per unit volume of scape was significantly greater than that of petioles (**Figure 10**), indicating that the former contained more cell walls than the latter. Niklas and Paolillo (1997) and Shah et al. (2017) reported that an increase in the number of cell walls per unit volume reflected strengths such as the bending strength, elastic modulus, and plastic deformation of the organ. Moreover, plants have different types of cells and tissues, and there are tissues that support the plant structure: the epidermis of the petiole is smooth and has a slightly thick cuticle; however, the outer layer of the cortical layer inside the epidermis is mainly sclerotic and also it is extensive, while the inner zone is composed of thin-walled parenchyma, which has flexible cellulose cell walls (Karam and Gibson, 1994). Kohler and Spatz (2002) showed that the outer strengthening tissues have an elastic modulus and strength approximately four times higher than the core tissues. In addition, the relative amounts of these tissues may play an important role in petiole flexibility during bending or standing under strong pressure; however, it is very difficult to measure the exact properties of different tissues and cell types (Karam and Gibson, 1994). Our anatomical results using toluidine blue O staining showed that the vascular bundle arrangement was more circular in scapes than in petioles, with blue-stained xylem arranged more regularly than in petioles (**Figure 12**). These tissues contain lignin in their walls, which cannot be bent or broken, and the relative amount of these tissues determines whether the plant will bend or stand under strong pressure (Moysset and Simón, 1991; Paiva and Machado, 2003; Leroux, 2012). Therefore, the scape of *F. japonicum* var. *japonicum* achieved a greater strength than the petiole by increasing the number of cell walls and arranging a pattern of supportive tissues. Although the period when the scapes are exposed to strong winds on the ground is shorter than that of leaves, the former were shown to be stronger than the latter and maintained their structure throughout the flowering period, from October to December.

*F. japonicum* var. *japonicum* is a cold-resistant evergreen perennial species (Koyama, 1995), and its leaves are continuously exposed to wind stress for long periods. The capitulum of *F. japonicum* var. *japonicum* consists of female ray flowers on the outside and bisexual tubular flowers in the center. The capitulum has yellow blooms on the long scape from autumn to winter that makes fluff like dandelions after flowering (Koyama, 1995). The period of exposure of the scapes to wind stress was shorter than that of the leaves. Nevertheless, why did the scape of *F. japonicum* var. *japonicum* need greater strength than the petiole even though it was shorter under strong winds like the petiole? Considering the surface area exposed to the wind, the area of capitula at the tips of *F. japonicum* var. *japonicum* are clearly smaller than that of the leaves (**Figure 1C**) and they do not receive as much drag from the wind as the leaves; therefore, it seems that something other than wind is involved in the stiffness of the scape. Attracting animal pollinators is essential for the reproductive success of the majority of angiosperm species, and flowers have been described as sensory billboards, catching the attention of potential pollinators and advertising the presence of nectar and pollen rewards within (Raguso, 2004). Capitulum of *F. japonicum* var. *japonicum* has been pollinated by bees, house flies, syrphids, and butterflies, and *Colletes perforator* Smith are identified as the pollinator involved in the hybridization of *F. japonicum* var. *japonicum* and *F. hiberniflorum* (Makino) Kitam (Yahara et al., 1986). These pollinators collect pollen while holding it on to flowers and move freely between flowers in the capitulum. Loads are placed on the scape when these pollinators are stationary or move within and among the capitulum, and even more so when many pollinators arrive simultaneously. Therefore, the scape of *F. japonicum* var. *japonicum* may be stiffer than the petiole to withstand not only wind stress, but also the sudden and unexpected load on the scape by pollinators, suggesting that these were reflected excessively in the strength of the scape of *F. japonicum* var. *japonicum*. *F. japonicum* var. *japonicum* could produce new leaves regardless of the season, even if this species loses its leaves due to strong winds; however, since scapes only develop above ground for a limited period of time, their loss due to strong winds has a significant impact on reproduction in that year, suggesting that the scapes not only have higher strength than the petioles, but also shorten them to reduce the bending moment caused by strong winds. In contrast, leaves attract herbivorous insects that add weight to the leaves. Leaves have been protected not only by direct defense against various chemical substances, but also by indirect defense, which recruits natural enemies and exterminates them through the diffusion of substances induced into the leaves by herbivorous insects (Karban et al., 1997; Agrawal and Rutter, 1998; Baldwin and Preston, 1999; Dicke et al., 2003). However, the larvae of the moth *Ostrinia* sp. fed on the lamina of *F. japonicum* var. *japonicum* because some of the chemical compounds in this species are sex pheromones for *Ostrinia* sp., as determined by analyses using gas chromatography coupled to a mass spectrometer and an electroantennographic detector (Tabata et al., 2008). Although wind stress was alleviated by the lamina area of *F. japonicum* var. *japonicum* being reduced, as the lamina was likely to be eaten, the weight of the larvae makes the leaves heavier; therefore, it will be interesting to elucidate the reproductive strategy under such conditions in the future. In addition, the large lamina of this species have the effect of reducing the wind speed around the individual, and so that the reduced wind speed had experienced by the flowers and inflorescences in populations with shortened scapes in coastal areas. Eaton et al. (2020) revealed that as the wind speed increased, the number of bees visiting flowers decreased significantly because the number of times bees hesitated to fly out of flowers increased significantly, and therefore, the microclimatic variation created by the large lamina may affect pollination in coastal populations with shortened scapes. Future research should take such points into consideration. In addition, Onoda et al. (2008) indicated that the mechanical properties of the leaf blade of *Plantago major* varied in different light environments. The comparison of mechanical properties between petiole, scape and leaf loss individuals under different photosynthetic conditions using e.g. forest floor ecotypes of *F. japonicum* is an interesting subject.

Our study has clarified the adaptation process of each aboveground organ in *F. japonicum* var. *japonicum* under strong wind stress in coastal areas. Morphological changes in plant species occur in the shape of individual roots when they had been subjected to certain mechanical stresses in the aboveground organs (Cannell and Coutts, 1988; Stokes et al., 1997). *F. japonicum* var. *japonicum* is a perennial herb (Koyama, 1995) and the size of its roots increases as it grows, allowing it to reproduce in the following year. Roots of *F. japonicum* var. *japonicum* also responds to the strong wind stress experienced by its aboveground organs in coastal areas. Our comparison of dry mass between the aboveground and below ground organs indicated that there was no significant difference between the coastal and inland populations of *F. japonicum* var. *japonicum* (**Figure 8**). This suggests that the effects of aboveground mechanical stress on *F. japonicum* var. *japonicum* did not differ between coastal and inland populations. Shiba et al. (2023) reported that in regions with higher wind speeds than those in this study, the aboveground morphology of *F. japonicum* var. *japonicum* tended to be smaller, suggesting potential impacts on below ground organs in these coastal populations. In contrast, *F. japonicum* var. *japonicum* typically grows on rocky substrates in areas with high wind velocities, making it difficult to collect individual below ground organs; thus, they could not be included in the measurements in this study.

Among the varieties of *F. japonicum*, *F. japonicum* var. *luchuense* could provide insights into the morphological, anatomical, and genetic mechanisms of local adaptation because the narrow leaves of *F. japonicum* var. *luchuense* was subjected to flash floods after heavy rain as a strong selective pressure in the area along rivers and streams, and was gained by the decreased number of leaf cells across the width of the leaf (Usukura et al., 1994). Plant groups including *F. japonicum* var. *luchuense* with such morphological leaf characteristics inhabiting riverbank habitats, are called rheophytes (van Steenis, 1981) and some previous studies have reported the convergent evolution of many phylogenetically distinct taxa of ferns and angiosperms (Imaichi and Kato, 1992, 1993; Setoguchi and Kajimura, 2004; Yamada et al., 2011; Ueda et al., 2012; Yokoyama et al., 2012; Ohga et al., 2012a; Matsui et al., 2013; Kato, 2017; Shiba et al., 2021). It would be interesting to evaluate whether the adaptation of *F. japonicum* var. *luchuense* under water stress showed the same pattern as *F. japonicum* var. *japonicum* in response to strong winds. Furthermore, a recent study revealed that petioles of the rheophytic species *Osmunda lancea* Thunb. (Osmundaceae), exhibit flexibility by increasing the size of their supportive cells, which helps avoid strong water stress along rivers (Shiba and Fukuda, 2024). By considering the adaptation of plant species to physical stress, such as strong winds and strong flash floods, there is endless interest in the diversification process of *F. japonicum* group, including the giant *F. japonicum* var. *giganteum* in coastal areas, as mentioned previously.

## Data availability statement

The original contributions presented in the study are included in the article/**Supplementary Material**. Further inquiries can be directed to the corresponding author.

## Author contributions

MS: Writing – review & editing, Writing – original draft, Methodology, Investigation, Formal analysis, Funding acquisition. SA: Writing – review & editing, Methodology, Investigation. SH: Writing – review & editing, Methodology, Software, Investigation. TF: Investigation, Writing – review & editing, Writing – original draft, Funding acquisition.
