# Correction: Impact on the scape of *Farfugium japonicum* var. *japonicum* (Asteraceae) under strong wind conditions based on morphological and mechanical analyses

**DOI:** 10.3389/fpls.2025.1633304

**Published:** 2025-06-25

**Authors:** Masayuki Shiba, Shuma Arihara, Shiori Harada, Tatsuya Fukuda

**Affiliations:** ^1^ Graduate School of Integrative Science and Engineering, Tokyo City University, Setagata, Tokyo, Japan; ^2^ Department of Science and Engineering, Tokyo City University, Setagata, Tokyo, Japan

**Keywords:** *Farfugium japonicum*, mechanical properties, petiole, scape, wind conditions, thigmomorphogenesis

Equation 1 in the **Methods** section, paragraph 3 was erroneously given as “V_fresh_ = πab/4h”. The correct equation is “V_fresh_ = (πab/4) × h”.

The authors apologize for this error and state that this does not change the scientific conclusions of the article in any way. The original article has been updated.

