# Supplementary material for: Impact on the scape of Farfugium japonicum var. japonicum (Asteraceae) under strong wind conditions based on morphological and mechanical analyses
Source: Front Plant Sci. 2024 Aug 6;15:1407127. doi: 10.3389/fpls.2024.1407127 (PMC11333370; doi:10.3389/fpls.2024.1407127)
Supplement: Supplementary file 1 [file Table_1.docx]

Supplementary Material

Impact on the scape of *Farfugium japonicum* var. *japonicum* (Asteraceae) under strong wind conditions based on morphological and mechanical analyses

**Masayuki Shiba^1^*, Shuma Arihara^2^, Shiori Harada^1^, Tatsuya Fukuda^1^**

^1^Graduate School of Integrative Science and Engineering, Tokyo City University, 1-28-1 Tamazutsumi, Setagata, Tokyo 158-8557

^2^Department of Science and Engineering, Tokyo City University, 1-28-1 Tamazutsumi, Setagata, Tokyo 158-8557

*** Correspondence:**Corresponding Author: Masayuki Shiba
msykshiba48@gmail.com

Supplementary Table 1. For mechanical properties, the specimen size is shown.

| Petiole | | | | | | | |
| --- | --- | --- | --- | --- | --- | --- | --- |
| Number | Length of specimen (mm) | Span lengh (mm) | a (mm) | b (mm) | *E* (MPa) | *I* (mm^4^) | *EI* (Nmm^2^) |
| 1 | 92.55 | 90 | 5.02 | 4.61 | 27.21 | 24.14 | 656.88 |
| 2 | 132.72 | 130 | 7.98 | 7.81 | 17.38 | 186.61 | 3242.85 |
| 3 | 100.32 | 100 | 6 | 5.43 | 27.02 | 47.15 | 1274.32 |
| 4 | 112.58 | 110 | 6.5 | 6.42 | 36.73 | 84.43 | 3101.44 |
| 5 | 92.21 | 90 | 5.53 | 5.47 | 33.54 | 44.43 | 1489.91 |
| 6 | 71.35 | 70 | 4.27 | 4.19 | 37.85 | 15.42 | 583.58 |
| 7 | 113.49 | 110 | 6.87 | 6.84 | 12.74 | 107.92 | 1374.60 |
| 8 | 111.6 | 110 | 6 | 5.14 | 20.82 | 40.00 | 832.73 |
| 9 | 111.57 | 110 | 6.75 | 6.14 | 61.57 | 76.70 | 4721.89 |
| 10 | 111.93 | 110 | 6.79 | 6.68 | 31.11 | 99.35 | 3091.17 |
| 11 | 111.23 | 110 | 6.11 | 6.01 | 47.72 | 65.11 | 3106.75 |
| 12 | 112.21 | 110 | 5.46 | 5.24 | 37.48 | 38.56 | 1445.29 |
| 13 | 91.26 | 90 | 5.11 | 4.99 | 20.63 | 31.17 | 642.83 |
| 14 | 122.39 | 120 | 7 | 6.9 | 41.93 | 112.88 | 4733.04 |
| 15 | 101.38 | 100 | 6.13 | 6 | 41.22 | 65.00 | 2679.00 |
| 16 | 103.08 | 100 | 5.56 | 5.35 | 68.58 | 41.79 | 2866.23 |
| 17 | 111.84 | 110 | 7 | 6.77 | 59.33 | 106.62 | 6325.27 |
| 18 | 101.78 | 100 | 5.5 | 5.2 | 84.69 | 37.96 | 3214.95 |
| 19 | 99.82 | 100 | 7.5 | 6.55 | 68.50 | 103.46 | 7086.73 |
| 20 | 100.32 | 110 | 6.37 | 6.35 | 42.05 | 80.06 | 3366.64 |
| 21 | 100.09 | 100 | 5.85 | 5.66 | 32.83 | 52.07 | 1709.41 |
| 22 | 61.44 | 60 | 4.45 | 3.56 | 32.33 | 9.86 | 318.63 |
| 23 | 98.42 | 100 | 6.2 | 6.02 | 42.77 | 66.40 | 2839.82 |
| 24 | 61.43 | 60 | 3.7 | 3.69 | 39.05 | 9.13 | 356.35 |
| 25 | 114.01 | 110 | 6.77 | 7.14 | 46.23 | 120.96 | 5592.13 |
| 26 | 117.05 | 110 | 5.79 | 5.37 | 49.00 | 44.01 | 2156.59 |
| 27 | 92.1 | 90 | 5.41 | 4.77 | 63.73 | 28.82 | 1836.82 |
| 28 | 112.31 | 110 | 6.79 | 5.98 | 53.09 | 71.28 | 3784.04 |
| 29 | 112.58 | 110 | 5.87 | 5.77 | 61.05 | 55.35 | 3379.26 |
| 30 | 92 | 90 | 5.06 | 4.83 | 74.25 | 27.99 | 2078.06 |
| 31 | 92.92 | 90 | 5.27 | 4.39 | 72.02 | 21.89 | 1576.26 |
| 32 | 114.69 | 110 | 6.64 | 6.52 | 56.33 | 90.34 | 5088.86 |
| 33 | 91.85 | 90 | 5.06 | 4.9 | 67.22 | 29.22 | 1964.30 |
| 34 | 82.33 | 80 | 5 | 4.56 | 58.92 | 23.27 | 1371.19 |
| 35 | 81.88 | 80 | 4.5 | 4.23 | 39.89 | 16.72 | 666.91 |

| Scape | | | | | | | |
| --- | --- | --- | --- | --- | --- | --- | --- |
| Number | Length of specimen (mm) | Span lengh (mm) | a (mm) | b (mm) | *E* (MPa) | *I* (mm^4^) | *EI* (Nmm^2^) |
| 1 | 141.96 | 140 | 8.33 | 7.74 | 115.32 | 189.60 | 21864.41 |
| 2 | 81.94 | 80 | 5 | 5.03 | 200.92 | 31.24 | 6275.78 |
| 3 | 90.84 | 90 | 5.44 | 5.28 | 59.63 | 39.31 | 2343.98 |
| 4 | 114.48 | 110 | 7.3 | 7 | 64.78 | 122.91 | 7961.59 |
| 5 | 91.34 | 90 | 5.6 | 4.36 | 59.35 | 22.78 | 1352.11 |
| 6 | 131.32 | 130 | 8.24 | 8 | 141.13 | 207.09 | 29227.43 |
| 7 | 150 | 150 | 12.92 | 11.39 | 133.31 | 937.14 | 124933.82 |
| 8 | 90.38 | 90 | 5.05 | 5.02 | 134.22 | 31.36 | 4209.23 |
| 9 | 122.18 | 120 | 7.22 | 7.03 | 88.51 | 123.13 | 10898.06 |
| 10 | 93.56 | 90 | 5.2 | 5.04 | 63.66 | 32.68 | 2080.29 |
| 11 | 91.48 | 90 | 5.1 | 4.74 | 47.09 | 26.66 | 1255.49 |
| 12 | 148 | 140 | 11.15 | 10.4 | 103.87 | 615.67 | 63947.49 |
| 13 | 108 | 110 | 6.42 | 6.34 | 157.82 | 80.31 | 12674.43 |
| 14 | 153.35 | 150 | 9.14 | 8.66 | 85.03 | 291.39 | 24777.07 |
| 15 | 112.71 | 110 | 6.64 | 6.27 | 82.37 | 80.34 | 6617.60 |
| 16 | 111.67 | 110 | 7.43 | 7 | 78.69 | 125.10 | 9843.67 |
| 17 | 114.31 | 110 | 7 | 5.95 | 79.66 | 72.38 | 5765.52 |
| 18 | 152.27 | 150 | 9.61 | 9.419 | 79.41 | 394.19 | 31303.00 |
| 19 | 101.8 | 100 | 6.4 | 6.39 | 135.75 | 81.97 | 11127.59 |
| 20 | 147 | 150 | 11.07 | 11 | 133.72 | 723.26 | 96715.13 |
| 21 | 152.25 | 150 | 9.12 | 8.1 | 27.96 | 237.91 | 6651.25 |
| 22 | 73.74 | 70 | 4.2 | 4.06 | 119.75 | 13.80 | 1652.29 |
| 23 | 124.74 | 120 | 7.2 | 7.01 | 181.96 | 121.75 | 22152.84 |
| 24 | 110.15 | 110 | 6.83 | 6.23 | 88.56 | 81.07 | 7179.17 |
| 25 | 143.34 | 140 | 8.74 | 8.53 | 108.12 | 266.27 | 28790.23 |
| 26 | 121.27 | 120 | 7.47 | 7.32 | 203.98 | 143.82 | 29337.33 |
| 27 | 121.05 | 120 | 6.94 | 6.61 | 44.36 | 98.39 | 4364.65 |
| 28 | 113.05 | 110 | 6.64 | 5.6 | 86.07 | 57.24 | 4926.67 |
| 29 | 111.36 | 110 | 7.13 | 6.432 | 155.98 | 93.13 | 14526.40 |
| 30 | 93.87 | 90 | 6.84 | 5.74 | 32.28 | 63.50 | 2049.79 |
| 31 | 150.73 | 150 | 12.9 | 11.14 | 73.29 | 875.42 | 64155.31 |
| 32 | 92.83 | 90 | 5.47 | 5.32 | 139.50 | 40.43 | 5640.00 |
| 33 | 69.04 | 68 | 4.15 | 3.91 | 96.41 | 12.18 | 1173.99 |
